# E90 subunit vaccine protects mice from Zika virus infection and microcephaly

**DOI:** 10.1186/s40478-018-0572-7

**Published:** 2018-08-10

**Authors:** Xingliang Zhu, Chunfeng Li, Shabbir Khan Afridi, Shulong Zu, Jesse W. Xu, Natalie Quanquin, Heng Yang, Genhong Cheng, Zhiheng Xu

**Affiliations:** 1 State Key Laboratory of Molecular Developmental Biology, CAS Center for Excellence in Brain Science and Intelligence Technology, Institute of Genetics and Developmental Biology, Chinese Academy of Sciences, Beijing, China; 2 University of Chinese Academy of Sciences, Beijing, 100101 China; 3 Center for Systems Medicine, Institute of Basic Medical Sciences, Chinese Academy of Medical Sciences & Peking Union Medical College, Beijing, 100005 China; 4 Suzhou Institute of Systems Medicine, Suzhou, 215123 Jiangsu China; 5 Department of Virology, State Key Laboratory of Pathogen and Biosecurity, Beijing Institute of Microbiology and Epidemiology, Beijing, 100071 China; 6 CAS Key Laboratory of Infection and Immunity, Institute of Biophysics, Chinese Academy of Sciences, Beijing, 100101 China; 7 Department of Microbiology, Immunology and Molecular Genetics, University of California, Los Angeles, CA 90095 USA; 8 Parkinson’s Disease Center, Beijing Institute for Brain Disorders, Beijing, 100101 China

**Keywords:** Zika virus, E90 vaccine, Protection, Microcephaly, Mouse model

## Abstract

Zika virus (ZIKV) became a global threat due to its unprecedented outbreak and its association with congenital malformations such as microcephaly in developing fetuses and neonates. There are currently no effective vaccines or drugs available for the prevention or treatment of ZIKV infection. Although multiple vaccine platforms have been established, their effectiveness in preventing congenital microcephaly has not been addressed. Herein, we tested a subunit vaccine containing the 450 amino acids at the N-terminus of the ZIKV envelope protein (E90) in mouse models for either in utero or neonatal ZIKV infection. In one model, embryos of vaccinated dams were challenged with a contemporary ZIKV strain at embryonic day 13.5. The other model infects neonatal mice from vaccinated dams by direct injection of ZIKV into the developing brains. The vaccine led to a substantial reduction of ZIKV-infected cells measured in the brains of fetal or suckling mice, and successfully prevented the onset of microcephaly compared to unvaccinated controls. Furthermore, E90 could protect mice from ZIKV infection even at 140 days post-immunization. This work directly demonstrates that immunization of pregnant mice protects the developing brains of offspring both in utero and in the neonatal period from subsequent ZIKV infection and microcephaly. It also supports the further development of the E90 subunit vaccine towards clinical trials.

**Electronic supplementary material:**

The online version of this article (10.1186/s40478-018-0572-7) contains supplementary material, which is available to authorized users.

## Introduction

Zika virus (ZIKV) belongs to the *Flaviviridae* family, and was first isolated from a sentinel monkey in the Zika forest of Uganda in 1947 [10]. Only sporadic human infections were reported in Africa and Asia until 2007, when the first ZIKV outbreak was documented on the Yap Island of Micronesia [26]. Like most other flaviviruses, ZIKV is predominantly spread by female *Aedes* spp. mosquitoes [23]. However, there is evidence that ZIKV can also be spread by mother-to-child vertical transmission [4, 24], sexual activity [14], and blood transfusion [25]. Prior to 2010, only benign symptoms were reported in ZIKV patients, such as light fever, maculopapular rash, conjunctivitis, and arthralgia, with 80% of cases being completely asymptomatic. However, during the recent outbreak in the Americas and the Caribbean, more severe clinical outcomes began to emerge [12]. Investigations using both human samples and animal models showed that ZIKV not only induces a series of immunological reactions [23, 29], but also infects neuronal progenitor cells (NPCs), potentially causing congenital microcephaly [6, 21, 23] or fetal demise [24].

Currently, a large number of anti-ZIKV therapeutics are being developed. Many of these candidates have been shown to be effective both in vitro and in vivo, including 25-hydroxycholesterol and chloroquine, and drugs such as Sofosbuvir, BCX4450, NITD008 and 7-DMA are entering phase I clinical trials [2, 20, 22, 36]. Another approach is immune-based therapy. Antibodies targeting specific ZIKV components have been shown to be able to prevent ZIKV infection in vivo [7, 37, 41]. Interferon therapy has also been tested, but is controversial due to conflicting results in different cell systems [15].

Compared to the above methods, vaccination is considered a potentially safer and more effective approach to preventing ZIKV infection. Several vaccines developed using different platforms and targets have also advanced into phase I clinical trials [3, 11, 31]. However, none of these candidates have been tested for their ability to protect the fetus or infants born to infected mothers, especially from devastating sequelae such as microcephaly. Han et al. showed that co-administration of ZIKV and sera from mice immunized with a ZIKV envelope protein subunit vaccine (E90) reduced the lethality of the infection, protecting neonates from death [16]. However, we sought to directly explore the potential of this vaccine to confer protection from pregnant dams to offspring in utero and reduce ZIKV-associated complications.

Given the life-long disabilities that may result from microcephaly, including cognitive and motor deficits, it is critically important that we continue to explore effective anti-ZIKV strategies. Herein, we investigated the protective effects of the E90 vaccine against ZIKV using both prenatal and neonatal mouse models. We successfully demonstrated that maternal immunization with E90 protected offspring from ZIKV challenge and microcephaly both in utero and in the neonatal period compared to placebo controls. Moreover, about 140 days after the first immunization, the immunized mice still carried significant titers of anti-ZIKV IgG that protected them from an otherwise lethal challenge with the virus.

## Materials and methods

Detailed methods are provided as follows:

- MOUSE EXPERIMENTS

ICR mice were purchased from Beijing Vital River Laboratory Animal Technology Co., Ltd.. All animals were bred in our core animal facility. After infections with ZIKV, all animals were housed in the P2 biosafety laboratory.

For immunizations, female mice were inoculated *i.p.* with about 100 μl of E90 (50 μg/mouse) or PBS (as a placebo) with the antigen adjuvant AddaVax™ (50 μl/mouse). The mice were boosted with the same dose of vaccine 14 days after the first immunization, and were bled 2 weeks later for serological analysis. Later, dams were mated to sires of the same age. One batch was used to perform in utero experiments, and the other was allowed to give birth, with neonates then used for further experiments.

For the prenatal microcephaly model, approximately 1 μl of ZIKV virus stock (600 PFU/mouse) or culture medium was injected into the cerebral lateral ventricle of E13.5 CD (ICR) mouse brains and inspected after 5 days as described previously. For each pregnant dam, 1/3 to 1/2 of the littermates were injected. In the postnatal model, around 100 PFU/mouse or culture medium was injected into the middle zone between the bregma point and lambda point of suckling mice at P1 or P2. The mice were weighed every other day and were sacrificed and inspected at P10.

- METHOD DETAILS
- Viruses

ZIKV strains (GZ01, GenBank: KU820898 or FSS13025, Genbank: JN860885) used in this study were described in our previous work [9, 22].

- Generation of protein subunit vaccine

ZIKV recombinant E90 protein was produced as described previously [16]. Briefly, the gene fragment encoding the first 450 amino acids of the E protein from ZIKV strain FSS13025 was cloned into the pET28a vector, and then expressed in *Escherichia coli* BL21 (DE3). The recombinant protein was purified by Ni-NTA agarose, and was checked by SDS-PAGE and confirmed by Western blot using mouse anti-flavivirus antibody 4G2. The concentration of purified E90 protein was measured using the BCA protein assay kit.

- ELISA

Purified recombinant ZIKV E protein [7] (100 ng/well) was coated on ELISA plates at 4 °C for overnight. Serum from CD1 mice was serially diluted with PBS containing 1% BSA and added to the E protein-coated plate for 1 h at 37 °C. The plate was then washed with PBST and incubated with HRP-conjugated IgG for 1 h at 37 °C. After washing with PBST, the plate was incubated with TMB solution before adding stop solution. The OD450 was measured with a microplate reader, and the samples were defined as positive by an OD450 two times higher than the background.

- RNA isolation and qRT-PCR

Total RNA from serum was extracted with the EasyPure Viral DNA/RNA Kit (TransGen Biotech, Beijing). ZIKV copies were measured by real-time quantitative PCR (qRT-PCR) [18]. ZIKV primers were described previously [8].

- Plaque reduction neutralization test (PRNT)

The neutralizing antibody titers in serum were determined in BHK-21 cells via PRNT as described [1]. Briefly, the serial dilutions of serum in PBS were mixed with ZIKV and incubated at 37 °C for 1 h. The above mixture was then added to BHK-21 cells and incubated for 1 h at 37 °C before adding overlay media mixed with agar. Plaques were counted after 4 days, with the average number in the control group set as 0, representing no neutralization.

- Histology and immunohistochemistry

Brains were harvested at E18.5 and P10, then were fixed in 4% PFA. After 24 h, the brain tissues were dehydrated in 30% sucrose, and frozen in TFM (tissue freezing medium) for cryosections after another 24 h. Sections (thickness: 40 μm) were immunostained as described previously [22]. Briefly, sections were blocked at RT for 1 h, incubated with the first antibody at 4 °C overnight, washed 3 times with PBST, then incubated with the secondary antibody at RT for 1 h, followed by 3 washes. The antibodies used for immunostaining are listed in the Additional file 1: Table S1. Slices were imaged on a LSM 700 (Carl Zeiss) confocal microscope (10×/0.3, 20×/0.5, 25 °C, in air medium) using ZEN software. For Nissl staining, brain slices were stained with 0.1% toluidine blue for 15 min, dehydrated serially in 70, 96, and 99% ethanol (45 s twice each). Finally, slices were hyalinized by Xylene for 5 min before sealing with neutral balsam.

- QUALIFICATION AND STATISTICAL ANALYSIS

Images were qualified with ZEN (Blue edition), ImageJ or Imaris as described previously [22]. All data were analyzed by GraphPad software. Statistical evaluations were performed by Student’s unpaired *t*-test. Data were presented as the mean ± standard error of the mean (*p ≤ 0.05, **p ≤ 0.01, ***p ≤ 0.001). Survival curves were analyzed by the log-rank (Mantel-Cox) test. All the representative images shown in the paper were from at least three independent experiments.

- ADDITIONAL RESOURCES

E90 and purified recombinant ZIKV E protein are kind gifts from Dr. Cheng-Feng Qin at the Beijing Institute of Microbiology and Epidemiology.

- SUPPLEMENTAL INFORMATION

The reagents and primers used in this paper are listed in the supplementary table.

## Results

### ZIKV E protein-based vaccine E90 protects adult mice from ZIKV infection

The E90 subunit vaccine is composed of the first 450 amino acids of the ZIKV FSS13025 strain envelope (E) protein [16], representing 90% of the coding sequence and excluding the C-terminal transmembrane domain. E90’s amino acid sequence is fairly conserved, sharing about 99.8% similarity with other ZIKV strains. The gene segment was expressed in *Escherichia coli* and purified as described previously [16]. 7–8 week-old female CD-1 (ICR) immunocompetent mice were divided into three groups. One group was inoculated with E90 (50 μg/mouse) and the adjuvant AddaVax™ (50 μl/mouse) by the intraperitoneal route (*i.p.*). The other two groups were injected *i.p.* with PBS and the same quantity of adjuvant. The mice were given a single boost two weeks later. 14 and 28 days following the first immunization, serum was collected for ELISA assay and standard plaque reduction neutralization testing (PRNT). A selection of mice from the immunized group and one placebo group were infected with the ZIKV GZ01 strain (10^5^ PFU/mouse) via the *i.p.* route, and their viremia was measured at 1 day post-infection (dpi).

**Fig. 1:**
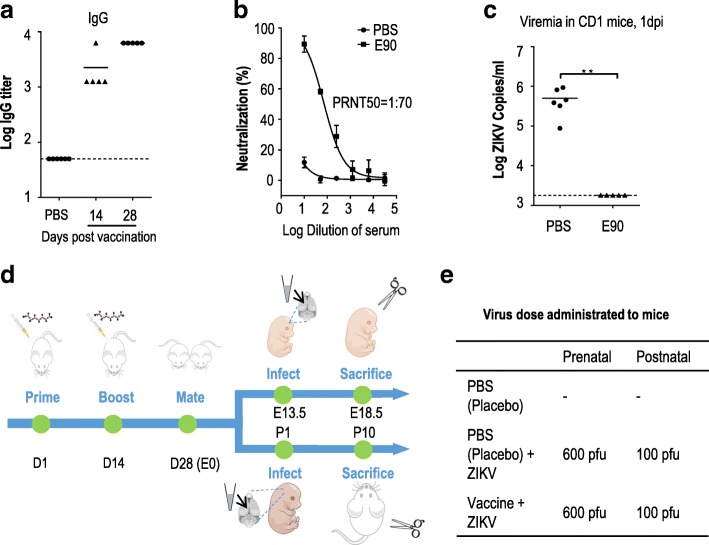
ZIKV E protein-based vaccine E90 prevents ZIKV infection in adult mice: **a.** ZIKV-specific IgG titers were analyzed at 14 and 28 days post-vaccination in mice. **b.** The ZIKV-neutralizing activity of serum from E90-immunized mice (2 weeks after a single boost) was analyzed by standard plaque reduction neutralization test (PRNT). **c.** ZIKV copies in serum from E90-immunized CD1 mice 1 day after ZIKV challenge (GZ01 strain, 10^5^ PFU/mouse) were analyzed by qRT-PCR. PBS, *n* = 6; E90, *n* = 5. All data are means ± SEM. Student’s *t*-test. **p < 0.01. **d.** Schematic for the immunizations of CD1 (ICR) female mice and the two models for testing the protection effect in offspring. **e.** Virus titers used in the prenatal and postnatal models

As shown in Fig. 1a-b, the specific anti-ZIKV IgG titer was as high as 10^4^ in the E90-immunized mice, with the PRNT_50_ titer from serum measured as 1:70, reflecting high titers of ZIKV-neutralizing antibodies. By contrast, mice vaccinated with PBS developed no detectable ZIKV-neutralizing antibodies or ZIKV-specific IgG (Fig. 1a-b). Consistent with these results, a significantly reduced viral burden was noted in the E90-vaccinated group compared to control mice after ZIKV challenge (Fig. 1c). Together, these results showed that the E90 subunit vaccine could effectively protect adult mice from ZIKV infection.

### E90 vaccine protects embryonic brains from ZIKV-induced microcephaly

**Fig. 2:**
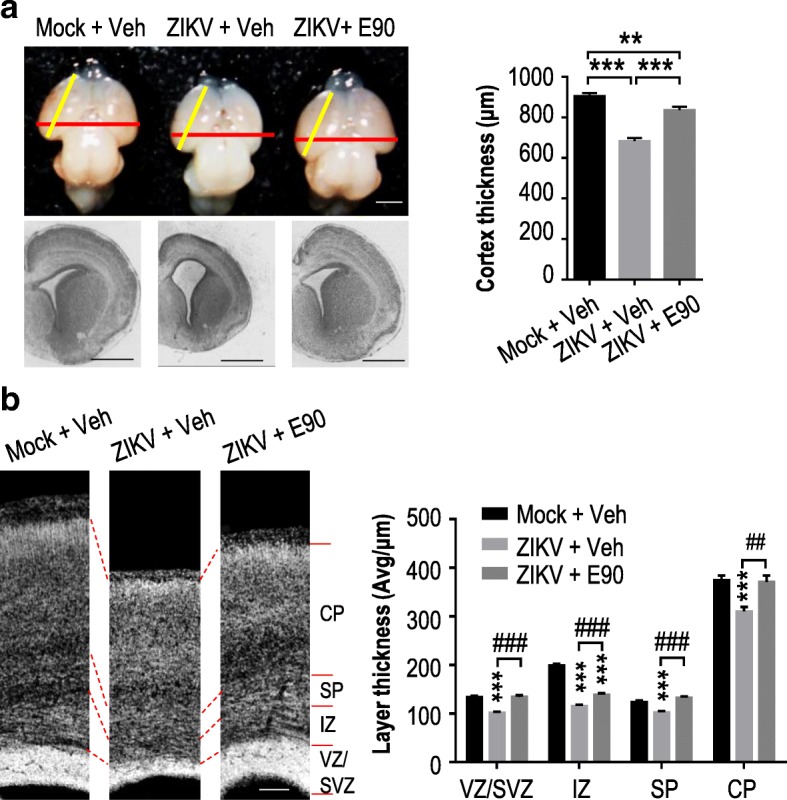
The E90 vaccine protects embryonic brains from ZIKV-induced microcephaly. **a.** Comparisons of brain sizes and cortex thickness between mock-infected mice (Mock + Veh), unvaccinated (ZIKV + Veh) and vaccinated (ZIKV + E90) infected groups. Embryonic brains were injected with ZIKV or medium at E13.5 and inspected at E18.5 as shown in Fig. 1d. Nissl staining of coronal sections is shown in the lower panel. Right panel shows measured cortex thickness. Mock + Veh: *n* = 12/4, ZIKV + Veh: *n* = 9/4, ZIKV + E90: n = 9/3. **b.** Nissl staining of different cortical layers. Right panel shows measured thickness of each layer. Mock + Veh: n = 12/4, ZIKV + Veh: n = 9/4, ZIKV + E90: n = 9/3. CP: cortical plate, SP: subplate, IZ: intermediate zone, SVZ: subventricular zone, VZ: ventricular zone. All data are means ± SEM. Student’s *t*-test. *p < 0.05, **p < 0.01, ***p < 0.001; ^##^*p* < 0.01, ^###^*p* < 0.001. ns: not significant. n: # of slices/# of individual brains. Scale bar = 1 mm (**a**), 100 μm (**b**)

**Fig. 3:**
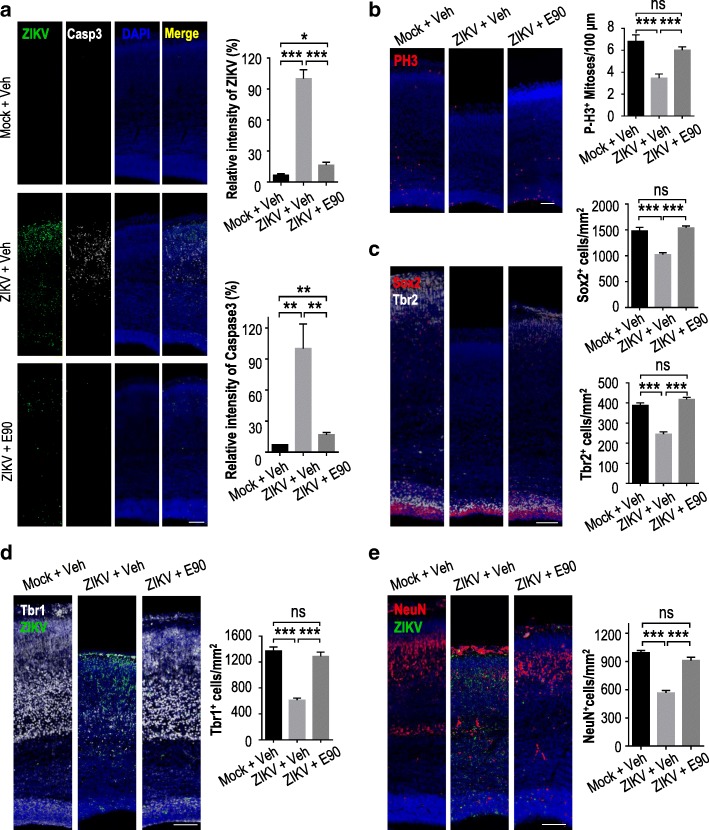
E90 protects embryonic brains from ZIKV infection, dysregulation of NPC proliferation, and loss of neurons. Mouse dams were vaccinated and embryos were infected as in Fig. 2. Coronal brain sections were stained with different antibodies as indicated. **a.** Relative signal intensity by fluorescent antibodies against ZIKV or the activated form of caspase3 (Casp3). Mock + Veh: n = 6/3, ZIKV + Veh: n = 6/3, ZIKV + E90: *n* = 7/3. **b.** Quantification of P-H3^+^ cell numbers in the cortices. Mock + Veh: *n* = 10/4, ZIKV + Veh: *n* = 8/4, ZIKV + E90: n = 9/3. **c.** Quantification of Sox2^+^ and Tbr2^+^ cell density. Mock + Veh: n = 10/4 (Sox2^+^, Tbr2^+^); ZIKV + Veh: n = 7/3 (Sox2^+^, Tbr2^+^); ZIKV + E90: n = 9/3 (Sox2^+^, Tbr2^+^). **d.** Quantification of Tbr1^+^ cell density in cortices. Mock + Veh: *n* = 11/4, ZIKV + Veh: n = 7/4, ZIKV + E90: n = 8/3. **e.** Quantification of NeuN^+^ cell density. Mock + Veh: n = 12/4, ZIKV + Veh: n = 8/4, ZIKV + E90: n = 8/3. All data are means ± SEM. Student’s *t*-test. *p < 0.05, **p < 0.01, ***p < 0.001. ns: not significant. n: # of slices/ # of individual brains. Scale bar = 100 μm (**a**-**e**)

We then evaluated the efficacy of this vaccine in protecting the offspring of pregnant mice from ZIKV infection, utilizing the same microcephaly model developed previously [21, 22, 39]. In this model, dams were given E90 or PBS as described above and mated at D28 (Fig. 1d). About 1 μl (600 PFU) of ZIKV or culture medium was injected into the lateral ventricle of fetal brains in utero at embryonic day 13.5 (E13.5) (Fig. 1e), as described previously [21]. Brain samples were inspected at E18.5. As shown in Fig. 2a, the average brain size of the vaccinated group was significantly larger than that of the placebo group. Likewise, the brain cortices of vaccinated mice remained much thicker after infection than those of infected unvaccinated mice (Fig. 2a). In addition, Nissl staining showed that individual cortical layers in the ZIKV-infected placebo group were significantly thinner than those in the mock and vaccinated groups (Fig. 2b). Furthermore, we inspected ZIKV density in embryonic brains via immunostaining, and found that the number of infected cells was considerably decreased in the vaccinated group compared to control mice (Fig. 3a). Similarly, the relative level of apoptosis (marked by activated caspase3^+^ cells) was significantly reduced in the vaccinated group (Fig. 3a).

ZIKV has been shown to infect neural progenitor cells (NPCs) and affect their proliferation [21, 35]. We therefore investigated the impact of vaccination on mouse NPCs by examining their levels of phosphorylated histone H3 (P-H3), a marker for being in the M-phase. We noticed comparable levels of P-H3^+^ cells in the cortices of mock-infected and infected vaccinated groups, with a reduction in infected unvaccinated mice (Fig. 3b). This same trend was seen when examining cortical cells for expression of Sox2^+^ (a NPC marker) and Tbr2^+^ (a marker for intermediate/basal progenitor cells) (Fig. 3c). In addition, the levels of Tbr1^+^ (a maker for immature neurons) and NeuN^+^ (a marker for mature neurons) cells were also both observed to be decreased in the infected unvaccinated group, compared to either the vaccinated or the uninfected groups (Fig. 3d-e). Together, these results demonstrate that E90 vaccination of mothers induces a protective immune response that is transmitted to fetuses to neutralize ZIKV, preventing the virus from replicating and inducing neuronal apoptosis or interfering with NPC proliferation, which could otherwise result in microcephaly.

### E90 vaccination of dams protects offspring from ZIKV-induced microcephaly

**Fig. 4:**
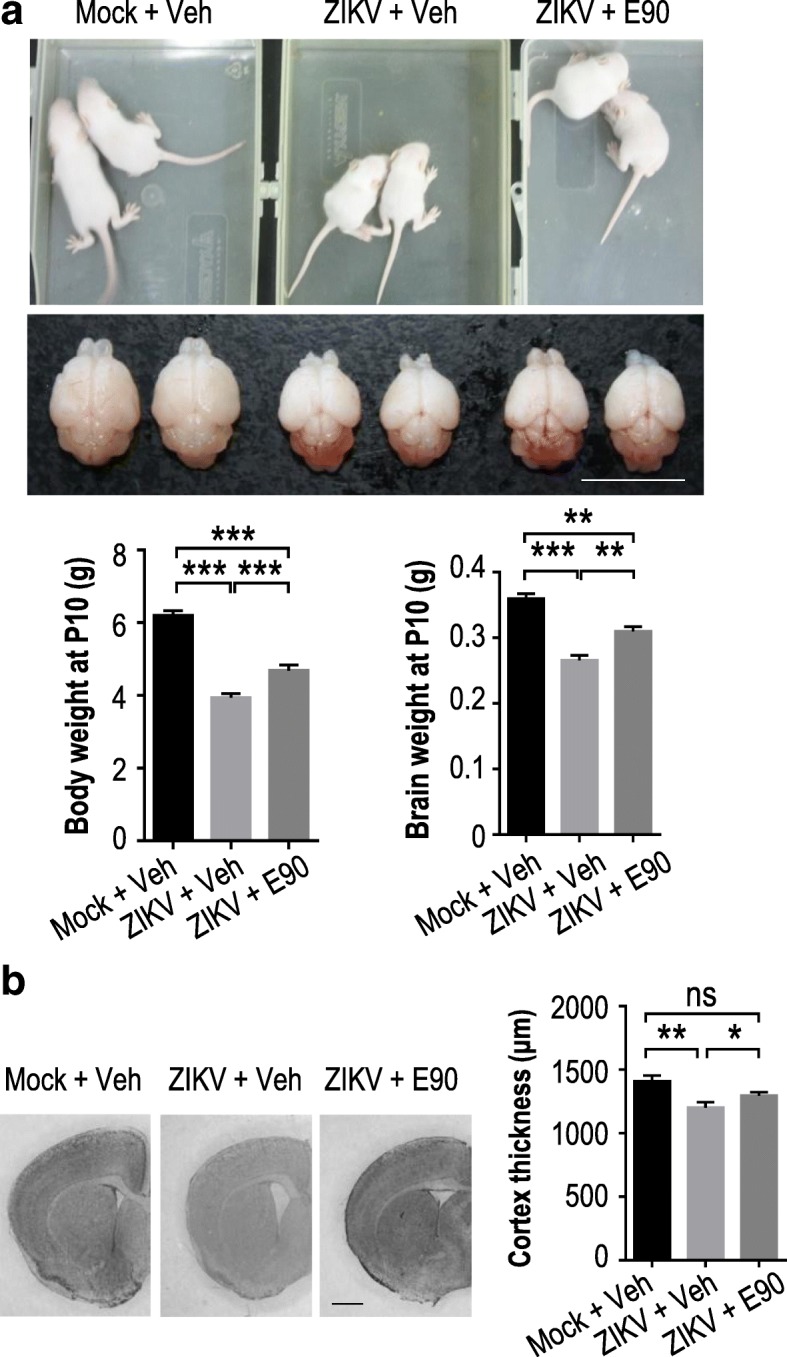
E90 vaccination of dams protects neonates from ZIKV-induced microcephaly. **a.** Comparison of body weights and brain sizes of mock–infected and ZIKV-infected vaccinated and unvaccinated groups as shown in Fig. 1d. Neonatal brains were injected with ZIKV or medium at P1 and inspected at P10. Lower panels: measurements of body and brain weights. For body weight, Mock + Veh: *n* = 15, ZIKV + Veh: n = 15, ZIKV + E90: *n* = 10. For brain weight, Mock + Veh: n = 5, ZIKV + Veh: n = 5, ZIKV + E90: *n* = 4. **b.** Nissl staining of coronal sections. Right panel: thickness of the cortical layer. Mock + Veh: n = 12/4, ZIKV + Veh: n = 8/3, ZIKV + E90: n = 11/4. All data are means ± SEM. Student’s *t*-test. *p < 0.05, **p < 0.01, ***p < 0.001. ns: not significant. n: # of slices/# of individual brains. Scale bar = 1 cm (**a**), 1 mm (**b**)

Although many infections are more likely to cause congenital defects when occurring in early stages of fetal development, cases of microcephaly from maternal ZIKV infections taking place late in the second trimester or even early in the third trimester have been reported [5, 17, 28]. As the gestation period of mice is relatively short, to simulate infections taking place in the later stages of human fetal development [13, 38], we injected the brains of one-day-old suckling mice with 100 pfu of ZIKV [40]. These mice were born to dams vaccinated with E90 or PBS (Fig. 1d), and we theorized that protective maternal antibodies may still be circulating or could be absorbed through the breast milk. The morbidity and mortality of these littermates were monitored. Pups in the placebo group showed significantly reduced weight gain in both their body and brain at P10 (9 days after ZIKV infection) compared to uninfected controls, while this effect was less pronounced in the vaccinated group (Fig. 4a). In addition, brain cortices of the neonatal mice in the vaccinated group were significantly thicker than those of the placebo group (Fig. 4b).

**Fig. 5:**
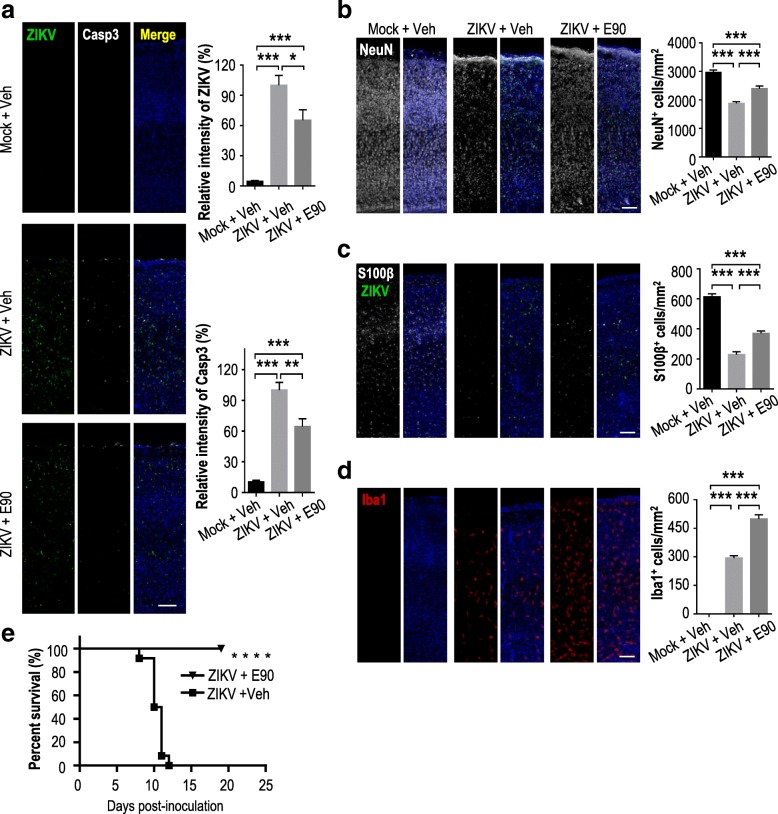
E90 vaccination of dams protects neonatal brains from ZIKV infection, cell death and survival. The brains of neonates born to vaccinated or unvaccinated mice were injected with ZIKV or medium as in Fig. 4. Coronal brain sections were stained with different antibodies as indicated. **a.** Relative signal intensity for ZIKV or activated caspase3 (Casp3). Mock + Veh: n = 11/4 (ZIKV), 11/4 (Casp3); ZIKV + Veh: n = 9/4 (ZIKV), 12/4 (Casp3); ZIKV + E90: n = 11/4 (ZIKV), 11/4 (Casp3). **b.** Quantification of NeuN^+^ cell numbers. Mock + Veh: n = 8/4, ZIKV + Veh: n = 8/4, ZIKV + E90: n = 9/4. **c.** Quantification of S100β^+^ cells. Mock + Veh: n = 11/4, ZIKV + Veh: n = 9/4, ZIKV + E90: n = 8/4. **d.** Quantification of Iba1^+^ cells. Mock + Veh: n = 11/4, ZIKV + Veh: n = 9/4, ZIKV + E90: n = 8/4. **e.** Improved survival rate of ZIKV-infected neonatal mice (P2) in the vaccinated group at 120 days post-vaccination. n = 12 per group, ****p < 0.0001, Log-rank (Mantel-Cox) test. Data (**a-d**) represent means ± SEM. Student’s *t*-test. *p < 0.05, **p < 0.01, ***p < 0.001. ns: not significant. n: # of slices/# of individual brains. Scale bar = 100 μm (**a**-**d**)

To more closely examine the level of infection in neonatal mouse brains, we did immunohistochemistry of cortical slices. We found that compared to the placebo group, the levels of ZIKV infection and cell death in different brain regions of neonatal mice born to vaccinated mothers were significantly decreased (Fig. 5a, Additional file 2: Figure S1). In addition, there was less reduction in mature neurons (NeuN as a marker) in the infected neonates born to vaccinated compared to unvaccinated mothers (Fig. 5b). Likewise, the density of S100β^+^ cells (a marker for glial progenitors) was significantly higher in the infected vaccinated group compared to the infected unvaccinated group (Fig. 5c), indicating that gliogenesis was preserved. Interestingly, activation of microglia (Iba1 as a marker) was noted in both infected groups, especially in mice born of vaccinated mothers (Fig. 5d), suggesting that the vaccine may have triggered a stronger immune response in those mice. In an additional experiment, we selected suckling neonatal mice born 120 days after dams had been vaccinated with E90 or PBS and challenged them 2 days later (P2) with a lethal dose of ZIKV. We noted that 100% of the mice born of immunized mothers survived, while there were no survivors in the placebo group (Fig. 5e). In summary, these results indicate that neonatal mice continue to benefit from the protective effects of maternal vaccination with E90, showing reduced destruction of neurons and glial cells caused by ZIKV infection. In addition, the duration of protection from the vaccine, as measured by improved survival against lethal ZIKV challenge in neonatal mice born of vaccinated dams, extends to at least 120 days.

### E90 protection from ZIKV infection extends to at least 140 days post-vaccination

**Fig. 6:**
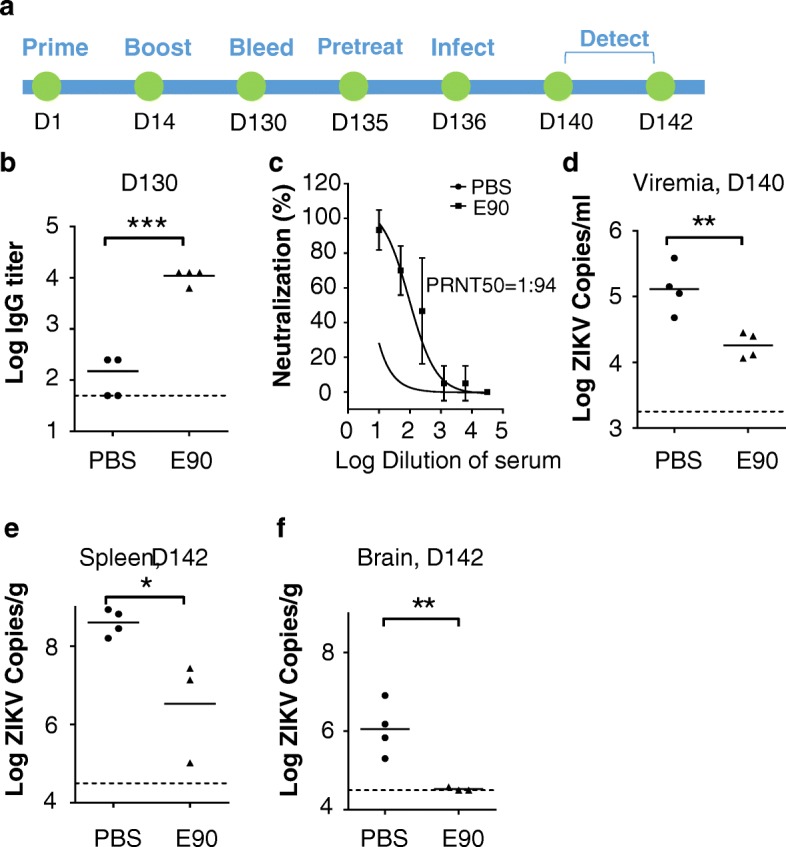
Vaccination with E90 protects mice against ZIKV infection for up to 140 days. **a.** Schematic for the immunizations of CD1 (ICR) female mice with subsequent testing between day 130 (D130) to day 142 (D142), treatment with anti-Ifanr1 antibody at D135, and ZIKV infection at D136. Viremia was measured at D140 and viral loads in organs were measured at D142. **b-c.** The anti-ZIKV titers were quantified by ELISA (**b**) or PRNT (**c**) in mice at D130. **d.** ZIKV viremia in mice measured at D140. **e-f.** Levels of ZIKV RNA recovered from the spleens (**e**) and brains (**f**) of ZIKV-infected mice. PBS, n = 4; E90, *n* = 3 or 4. All data are means ± SEM. Student’s *t*-test. *p < 0.05, **p < 0.01, ***p < 0.001

To further characterize the protective effects of E90 several months after vaccination, CD-1 female mice were immunized as above, and serum was collected at day 130 (D130) post-immunization for ELISA and PRNT assays (Fig. 6a). Immunized mice were then treated with anti-Ifnar1 antibody (2 mg/mouse) at D135 and challenged with ZIKV (GZ01 strain, 10^5^ PFU/mouse) via the *i.p.* route at D136. Viral loads in the sera of mouse dams were measured at D140. Two days later, the mice were sacrificed and viral RNA was isolated from the brains and spleens for measurement by qRT-PCR. Our results showed that at D130, anti-ZIKV IgG levels remained as elevated as they were 14 days after boost (Fig. 6b and Fig. 1a). The PRNT_50_ was estimated as 1:94 (Fig. 6c). As expected, viremia in vaccinated mice at D140 was largely reduced compared to unimmunized mice (Fig. 6d). Consistent with this result, viral loads in the spleens and brains of unimmunized mice were very high, while those of E90-vaccinated mice were significantly reduced (Fig. 6e-f). These results indicate that E90 elicits high titers of long-lasting ZIKV-neutralizing antibodies, which can protect immunocompetent mice from ZIKV infection up to at least 142 days after the first vaccine dose.

## Discussion

After ZIKV was discovered to be a global threat to public health, researchers began striving to develop effective therapeutics and interventions. Several vaccine candidates employing different strategies are currently undergoing clinical trials, such as Inovio’s DNA vaccine [11], the NIAID/Butantan pentavalent live-attenuated vaccine (ZIKV + DENV), and a ZIKV purified inactivated vaccine (ZPIV, NIAID/BARDA/WRAIR/Sanofi Pasteur/+Fiocruz) [23, 31, 36]. However, most of these ZIKV vaccine platforms were not evaluated for protective effects during pregnancy. Although Richner et al. did look at the effectiveness of the modified mRNA and the live-attenuated candidates against in utero transmission of ZIKV in mice [32], they did not show direct evidence of their protective roles in embryonic and neonatal brains after ZIKV infection, including microcephaly-related complications.

Research to create a ZIKV vaccine took inspiration from previous efforts to develop a vaccine for dengue virus (DENV), a related flavivirus. A peptide derived from the DENV E protein has been reported to inhibit infection by DENV types 1–4 and even other flaviviruses, including Central European encephalitis virus, Russian Spring-Summer encephalitis virus and West Nile virus [33]. E protein is a reasonable vaccine target due to its critical roles in viral attachment, entry, and replication in the infected host. Neutralizing antibodies produced in vivo against the E protein work well against other flaviviruses, such as the Japanese encephalitis, yellow fever, and tick-borne encephalitis viruses [30]. In 2016, Larocca et al. developed a prM-Env DNA vaccine offering complete protection against ZIKV [19]. Diamond et al. demonstrated that an mRNA vaccine encoding prM-E protected mice from ZIKV infection [32]. Pardi et al. further showed that a prM–E mRNA vaccine enveloped by lipid-nanoparticles protected non-human primates from ZIKV infection even 5 weeks after immunization [27]. In 2017, Han et al. found that E90 antisera from immunized mice could directly neutralize ZIKV by co-administering the sera and a lethal dose of ZIKV together *i.p.* into neonatal mice, who showed improved survival over infected mice [16]. Our study has expanded upon this work, demonstrating for the first time that the protective effects of a ZIKV vaccine in mouse dams can be transmitted transplacentally to offspring, leading to improved survival and a reduction in ZIKV burden and impairment of neural cells after in utero or perinatal ZIKV infection compared to infected mice born to unvaccinated mothers.

To overcome the shortcomings of existing vaccines and expand our knowledge of ZIKV-related effects in the embryonic period, we adopted the E90 subunit vaccine platform for use in two mouse microcephaly models. One model inspects the brain at E18.5 (5 days after ZIKV inoculation), which helps us to investigate the protective effect in the embryonic period. The other examines the brain at P10, 9 days following ZIKV infection of neonatal mice, which we suggest could be comparable to late trimester infections in humans. Our study demonstrated the efficacy of the E90 vaccine in reducing the viral burden and impaired development of ZIKV-infected mouse brains in both models. Additionally, to represent even earlier time points in development, we infected mice at E6, which we suggest might be the equivalent to the first trimester in humans. We found that at E16.5, the viral load in the placenta from the group whose mothers were vaccinated with E90 was significantly reduced compared to the PBS group (Additional file 3: Figure S2a-b). Moreover, there were clear signs of damage to the fetus in the placebo group at E18.5, compared to the mice born of vaccinated mothers, who all delivered normally at E20 (Additional file 3: Figure S2c-d).

We also inspected the safety of this vaccine and found no obvious adverse effects in mice, including changes in body weight (Additional file 4: Figure S3). Since this is a protein-based vaccine, there is no risk of virulence or concerns for mutant reversion, oncogenic insertions, or other complications related to live vaccines or nucleic acid vaccine platforms. Safety is a critical concern for a ZIKV vaccine, given that both pregnant women and their developing infants—the population most devastated by this infection, are immunocompromised. Other safety concerns with live vaccine strains are that they might be taken up by mosquitos and recombined with wild-type strains, although Shan et al. specifically tested their attenuated ZIKV strain and found it was incapable of infecting mosquitoes [34]. A cost for protein vaccines being non-infectious and considered relatively safe is their low immunogenicity, which may require multiple doses or the use of an adjuvant to stimulate effective neutralizing antibody titers. After a single boost of E90 combined with the AddaVax™ adjuvant in adult female mice, we measured the serum anti-ZIKV IgG titer to be approximately 10^4^, which persisted for at least 116 days after the boost (Fig. 1a and Fig. 6b). This represents long-lasting protection against ZIKV infection, which we demonstrated through reduced viremia and organ burdens in those mice after viral challenge.

Our findings in adult, embryonic and neonatal mice using the E90 vaccine are promising, however we acknowledge that the protection was not complete. The ZIKV burden and neuronal cell death were reduced in vaccinated mice compared to unvaccinated controls, but not completely eliminated. Gliogenesis was also more prevalent in the vaccinated group, but still reduced compared to uninfected controls.

In conclusion, we were able to demonstrate that the E90 subunit vaccine could confer protection to adult mice against ZIKV as measured by the reduced viral burden in the brain, and that this protection was also seen in their offspring using models of infection at different stages of development. Our studies demonstrate the importance of testing ZIKV vaccines in pregnant animal models, and show evidence that this protection can significantly preserve fetal brain development and reduce the incidence and extent of microcephaly.

## Conclusions

We demonstrate the E90 subunit vaccine can protect adult mice from ZIKV infection. Immunization of pregnant dams protects their fetuses and offspring from ZIKV infection and reduces the risk of microcephaly. Moreover, the protective effect of E90 against ZIKV is present even 128 days after the second dose.

## Additional files

Additional file 1: Supplemental experimental materials. (XLSX 10 kb)

Additional file 2: **Figure S1.** E90 suppresses ZIKV infection in different regions of neonatal mouse brains. a-e. Comparisons of ZIKV infection and apoptosis in mock–infected or ZIKV-infected vaccinated and unvaccinated groups. Neonatal pup brains were injected with ZIKV (100 PFU/mouse) or medium at P1 and inspected at P10. Brain sections were stained with antibodies for ZIKV (green) or the activated form of Caspase3 (white). a: Hippocampus, b: Striatum, c: Thalamus, d: Hypothalamus, e: Cerebellum. Scale bar = 200 μm (a-e). (PDF 194 kb)

Additional file 3: **Figure S2.** Vaccination of female mice protects offspring from early ZIKV infection. **a.** Schematic for immunizations and mating of CD1 (ICR) female mice with ZIKV challenge in early pregnancy. Mice vaccinated with E90 or PBS were mated at D28 and treated with anti-Ifnar1 antibody at E5.5. Mice were infected with ZIKV virus (GZ01 strain) at E6.5. **b.** Viral loads in placenta at E16.5 were measured by qRT-PCR. *n* = 16 for each group. All data are means ± SEM. Student’s *t*-test. ***p < 0.001. **c.** Condition of embryos in the PBS group after ZIKV infection at E18.5 (arrows indicate physical deformities, also shown in the left lower image). Scale bar = 0.5 cm **d.** Newborn mice at P1 born to E90-vaccinated mothers. Scale bar = 1 cm. (PDF 92 kb)

Additional file 4: **Figure S3.** E90 vaccination does not affect body weight significantly. Body weights of vaccinated or control mice before mating. Mock + Veh: *n* = 5, Mock + E90: *n* = 5. Data are means ± SEM. Student’s *t*-test. ns: not significant. (PDF 70 kb)
