# Supplementary figures and images for: E90 subunit vaccine protects mice from Zika virus infection and microcephaly
Source: Acta Neuropathol Commun. 2018 Aug 10;6:77. doi: 10.1186/s40478-018-0572-7 (PMC6086021; doi:10.1186/s40478-018-0572-7)

**Figure S1**

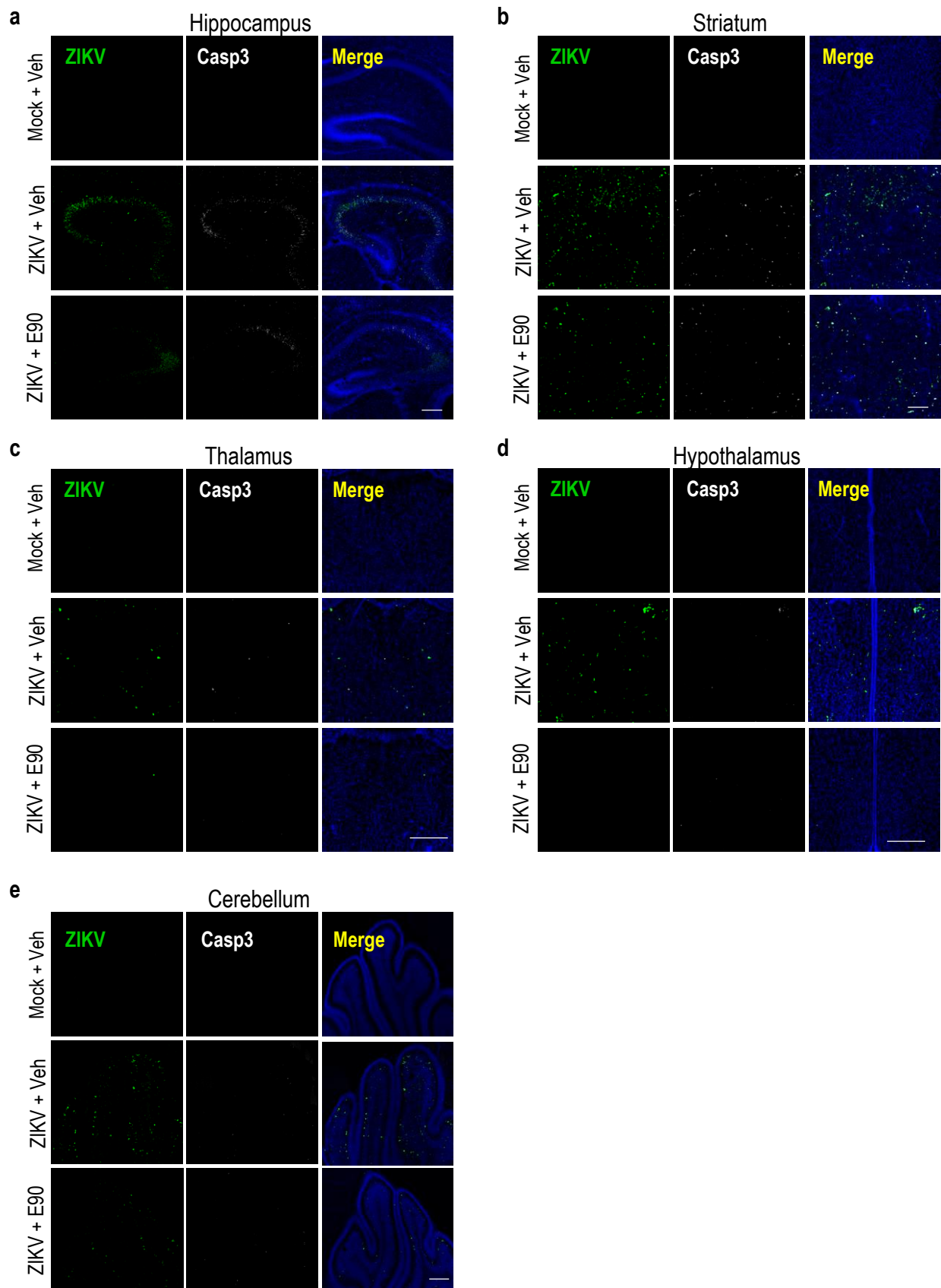

Supplement: Supplementary file 2 — Figure S1. E90 suppresses ZIKV infection in different regions of neonatal mouse brains. a-e. Comparisons of ZIKV infection and apoptosis in mock–infected or ZIKV-infected vaccinated and unvaccinated groups. Neonatal pup brains were injected with ZIKV (100 PFU/mouse) or medium at P1 and inspected at P10. Brain sections were stained with antibodies for ZIKV (green) or the activated form of Caspase3 (white). a: Hippocampus, b: Striatum, c: Thalamus, d: Hypothalamus, e: Cerebellum. Scale bar = 200 μm (a-e). (PDF 194 kb) [file 40478_2018_572_MOESM2_ESM.pdf]

Figure S2

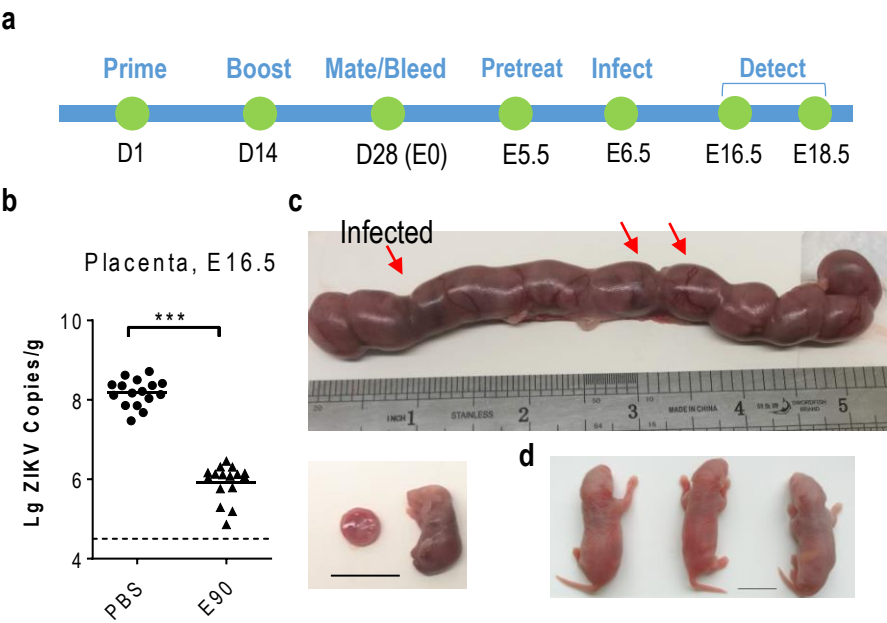

Supplement: Supplementary file 3 — Figure S2. Vaccination of female mice protects offspring from early ZIKV infection. a. Schematic for immunizations and mating of CD1 (ICR) female mice with ZIKV challenge in early pregnancy. Mice vaccinated with E90 or PBS were mated at D28 and treated with anti-Ifnar1 antibody at E5.5. Mice were infected with ZIKV virus (GZ01 strain) at E6.5. b. Viral loads in placenta at E16.5 were measured by qRT-PCR. n = 16 for each group. All data are means ± SEM. Student’s t-test. ***p < 0.001. c. Condition of embryos in the PBS group after ZIKV infection at E18.5 (arrows indicate physical deformities, also shown in the left lower image). Scale bar = 0.5 cm d. Newborn mice at P1 born to E90-vaccinated mothers. Scale bar = 1 cm. (PDF 92 kb) [file 40478_2018_572_MOESM3_ESM.pdf]

Figure S3

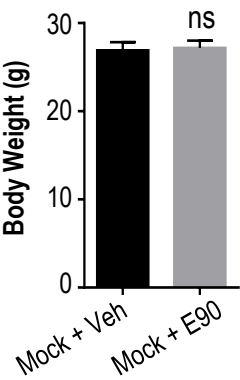

Supplement: Supplementary file 4 — Figure S3. E90 vaccination does not affect body weight significantly. Body weights of vaccinated or control mice before mating. Mock + Veh: n = 5, Mock + E90: n = 5. Data are means ± SEM. Student’s t-test. ns: not significant. (PDF 70 kb) [file 40478_2018_572_MOESM4_ESM.pdf]
